# Sub-acute administration of (*S*)-dimethyl 2-(3-(phenyltellanyl) propanamido) succinate induces toxicity and oxidative stress in mice: unexpected effects of N-acetylcysteine

**DOI:** 10.1186/2193-1801-2-182

**Published:** 2013-04-24

**Authors:** Daiane F Meinerz, Bruna Comparsi, Josiane Allebrandt, Douglas Oscar Ceolin Mariano, Danúbia B dos Santos, Ana Paula Pegoraro Zemolin, Marcelo Farina, Luiz Alcir Dafre, João B T Rocha, Thaís Posser, Jeferson L Franco

**Affiliations:** Departamento de Química, Centro de Ciências Naturais e Exatas, Universidade Federal de Santa Maria, Santa Maria, RS CEP 97105-900 Brazil; Universidade Federal do Pampa, Campus São Gabriel, São Gabriel, RS CEP 97300-000 Brazil; Departamento de Bioquímica, Centro de Ciências Biológicas, Universidade Federal de Santa Catarina, Florianópolis, SC CEP 88040-900 Brazil

## Abstract

The organic tellurium compound (S)-dimethyl 2-(3-(phenyltellanyl) propanamide) succinate (TeAsp) exhibits thiol-peroxidase activity that could potentially offer protection against oxidative stress. However, data from the literature show that tellurium is a toxic agent to rodents. In order to mitigate such toxicity, N-acetylcysteine (NAC) was administered in parallel with TeAsp during 10 days. Mice were separated into four groups receiving daily injections of (A) vehicle (PBS 2.5 ml/kg, i.p. and DMSO 1 ml/kg, s.c.), (B) NAC (100 mg/kg, i.p. and DMSO s.c.), (C) PBS i.p. and TeAsp (92.5 μmol/kg, s.c), or (D) NAC plus TeAsp. TeAsp treatment started on the fourth day. Vehicle or NAC-treated animals showed an increase in body weight whereas TeAsp caused a significant reduction. Contrary to expected, NAC co-administration potentiated the toxic effect of TeAsp, causing a decrease in body weight. Vehicle, NAC or TeAsp did not affect the exploratory and motor activity in the open-field test at the end of the treatment, while the combination of NAC and TeAsp produced a significant decrease in these parameters. No DNA damage or alterations in cell viability were observed in leukocytes of treated animals. Treatments produced no or minor effects on the activities of antioxidant enzymes catalase, glutathione peroxidase and glutathione reductase, whereas the activity of the thioredoxin reductase was decreased in the brain and increased the liver of the animals in the groups receiving TeAsp or TeAsp plus NAC. In conclusion, the toxicity of TeAsp was potentiated by NAC and oxidative stress appears to play a central role in this process.

**Electronic supplementary material:**

The online version of this article (doi:10.1186/2193-1801-2-182) contains supplementary material, which is available to authorized users.

## Introduction

Elemental tellurium (Te) is a rare trace element that is widely used in the manufacture of ceramics, glass, semiconductors, and metals (Ogra et al. 2008). Despite the growing use of organotellurium compounds in chemistry and biochemistry and the consequent increase in the risk of occupational and environmental exposure to these substances, data about their toxicity are scarce in the literature. In fact, these compounds have been shown to be promising and useful alternatives for numerous synthetic operations in organic synthesis (Petragnani 1994; Comasseto and Gariani 2009). Previous studies demonstrated that organotellurium compounds are potentially toxic and lethal to rodents at low doses (Meotti et al. 2003; Savegnago et al. 2006) Indeed, tellurides can cause cytotoxicity (Sailer et al. 2004), hepatotoxicity (Meotti et al. 2003), neurotoxicity (Nogueira et al. 2001; Nogueira et al. 2002), teratogenicity (Stangherlin et al. 2005) and genotoxicity (Santos et al. 2009a, b). Moreover, these compounds can inhibit sulfhydryl-containing enzymes, such as the Na^+^/K^+^-ATPase (Borges et al. 2005), the δ-aminolevulinic acid dehydratase (Maciel et al. 2000; Nogueira et al. 2003) and the squaleno monooxigenase (Laden and Porter 2001).

The mechanisms of toxicity by organotellurium compounds may be related to the oxidation of thiol groups of important biomolecules (Nogueira et al. 2004), the replacement of selenium in selenoproteins (such as thioredoxin reductase) (Engman et al. 2000), and the capacity of Te compounds to induce the formation of reactive oxygen species (ROS) (Chen et al. 2001; Funchal et al. 2011; de Andrade et al. 2010). On the other hand, pharmacological and/or antioxidants properties of tellurium compounds have also been reported in the literature (Ávila et al. 2011; Avila et al. 2012), including antitumor and chemoprotective effects (Engman et al. 2000; Cunha et al. 2005), and glutathione peroxidase (GPx) like activity (Engman et al. 1994). The existence of toxic and beneficial effects of tellurium compounds brings out the need for further research on their toxicological and pharmacological mechanisms of action.

The compound studied on the present work, *(S*)-dimethyl 2-(3-(phenyltellanyl) propanamido) succinate (TeAsp), is a new telluroamino acid derivative of aspartic acid that was found to be the most potent mimetic of GPx among 20 newly synthesized derivatives (Braga et al. 2009; Alberto et al. 2009) presenting antioxidant properties *in vitro*. However, when administered to rodents this compound showed potent genotoxic and mutagenic actions (Meinerz et al. 2011).

In order to elucidate the mechanisms mediating the biological effects of TeAsp, the present study aimed to investigate putative mechanisms underlying the toxicity of TeAsp *in vivo*. We assessed behavioral and oxidative stress parameters in mice, including the modulation of antioxidant enzymatic defense systems by TeAsp in different tissues. We also evaluated the potential protective effect of N-acetylcysteine (NAC), a thiol-containing antioxidant that is largely used to mitigate the effects of oxidative stress-causing agents (Flora et al. 2004) due to its ability to stimulate glutathione (GSH) synthesis.

## Materials and methods

### Chemicals

**Figure 1:**
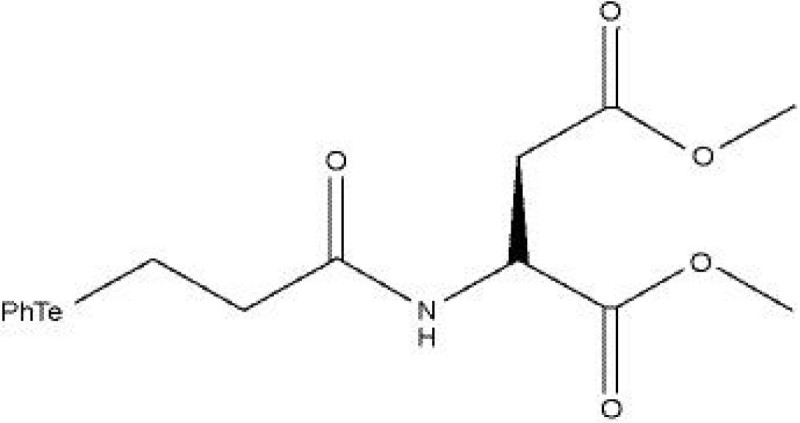
**The chemical structure of (** ***S*** **)-dimethyl 2-(3-(phenyltellanyl) propanamido) succinate (TeAsp).**

The chemical structure of TeAsp is shown in Figure 1. This compound was synthesized by the method previously described by Braga et al. (2009). TeAsp solutions were prepared in dimethyl sulfoxide (DMSO). All reagents used were purchased from Sigma (São Paulo, Brazil).

### Animals

Swiss adult male mice (45-days old), weighing 30–40 g, were used in the experiments. All mice were obtained from and maintained in the animal facilities of UFSM. Mice (5 per group) were kept in separate cages on 12-hour light/dark cycle, controlled temperature (22±3°C) and *ad libitum* access to food and water. The animals were used according to the guidelines of the institutional board for animal care and use (CEUA) of the Federal University of Santa Maria, Brazil (23081.002435/2007-16).

### Exposure (animals treatment)

Mice were separated into four groups, with 5 animals each, receiving daily injections of: (A) vehicles of NAC and TeAsp respectively (PBS 2.5 ml/kg, i.p. plus DMSO 1 ml/kg (0.1%), s.c.); (B) NAC (100 mg/kg, i.p.) plus DMSO s.c.); (C) PBS i.p. plus TeAsp (92.5 μmol/kg, s.c.); or (D) NAC, i.p plus TeAsp, s.c. Treatment with TeAsp started just after the third day of NAC administration, using a dose equivalent to 50% of the previously described LD_50_ (185 μmol/kg) (Meinerz et al. 2011).

The animals were weighted daily and monitored for the appearance of signs of toxicity, such as exhalation of garlic odor, partial or total paralysis of lower limbs, diarrhea, tremors, hair loss, and weight loss. No signs of toxicity were apparent throughout the period of treatment. Behavioral evaluation was performed 24 hours after the end of the treatment.

### Open field test

One day after the last injection the animals were acclimated to the experimental room for at least 2 hours prior to the beginning of the open field test. Open field tests were carried out in soundproof room without any human interference, as described elsewhere (Kim et al. 2000).

### Antioxidant enzymes assays and protein determination

On the 11^th^ day, following the behavior evaluation, the animals were anesthetized with ethyl ether and blood samples were collected by heart puncture for leukocytes viability and comet assay. The mice were then euthanized; the brain and liver were dissected, weighted, homogenized in a Potter- Elvehjem tissue homogenizer with 50 mM Tris–HCl buffer, pH 7.4 and centrifuged at 4000 × g for 10 min at 4°C. The low speed supernatant (S1) was collected and kept on ice until used for the enzyme assays.

The activities of the antioxidant enzymes catalase (CAT) (Aebi 1984), GPx (Wendel 1981), glutathione reductase (GR) (Carlberg and Mannervik 1985) and thioredoxin reductase (TrxR) (Holmgren and Björnstedt 1995) were determined in the S1 fraction of mice brain and liver as described elsewhere.

Protein concentration was determined by the method of Lowry et al. (1951), using bovine serum albumin as the standard.

### Leukocyte DNA damage and viability

Leukocytes were isolated as describes in (Santos et al. 2009a) and the cell density was adjusted to 2 × 10^6^ leukocytes/ml by dilution with HBSS/heparin. The percentage of viable leukocytes was determined using the Trypan blue exclusion method counting on a hemocytometer (Mischell and Shiingi 1980).

The comet assay was carried out following previously published procedures (Santos et al. 2009a). The slides were analyzed by at least two observers blinded to the experiment. One hundred cells per sample were randomly selected and visually scored based on tail intensity into five classes ranging from 0 (undamaged) to 4 (maximally damaged). The damage index (DI) is based on the length of migration and on the amount of DNA in the tail was calculated as follows:

DI=n1+2n2+3n3+4n4

where, n1 represents the number of cells with level 1 of damage; n2, number of cells with level 2 of damage; n3, number of cells with level 3 of damage; n4, number of cells with level 4 of damage.

### Statistical analysis

Data were analyzed using one or two way analysis of variance (ANOVA) followed by Newman-Keuls or Bonferroni post hoc test when appropriate. Differences were considered significant when p<0.05.

## Results

### Body weight gain

**Figure 2:**
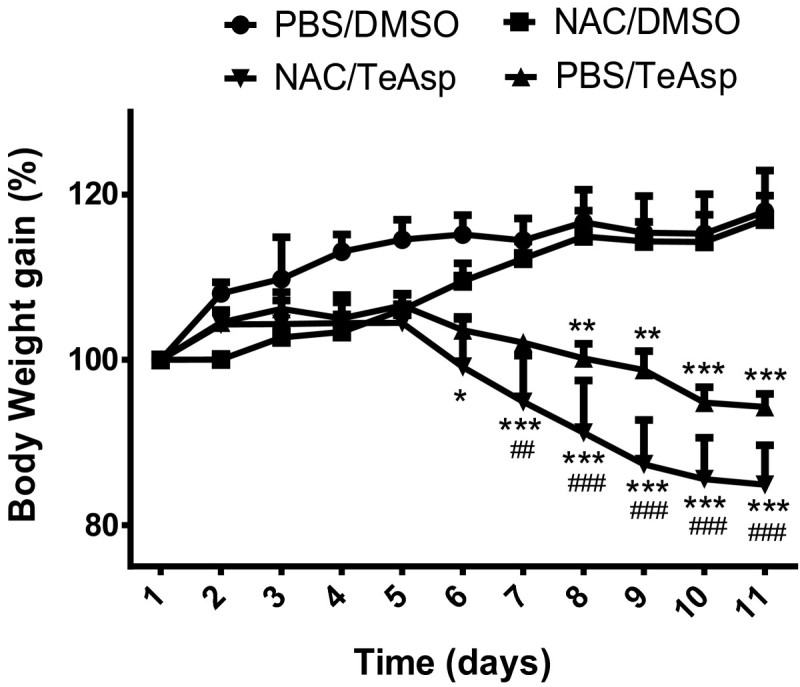
**Effect of (*S*)dimethyl 2-(3 (phenyltellanyl) propanamido) succinate (TeAsp) on the body weight gain of adult mice.** Data are reported as percentage ± S.E.M. of 5 animals per group. Two way ANOVA followed by Bonferroni posttest. * (p<0.05), ** (p<0.01) or *** (p<0.001) represent significant differences from PBS/DMSO and ^##^ (p<0.01) or ^###^ (p<0.001) means significant differences from NAC/DMSO group.

No mortality occurred after repeated administration of NAC and/or TeAsp to mice. As expected, the animals’ body weight gains increased (17%) with time (Figure 2). Treatment with TeAsp for seven days (days 4–10) caused a significant decrease in body weight gain already in the fourth day after the first administration (day 7), producing a continuous decrease until the end of the experiment, when animals weighted 6% less. Animals treated with NAC followed the same trend. Compared to control (vehicle-treated) animals at the end of the treatment TeAsp-treated mice presented an average 23% lower body weight. Instead of decreasing TeAsp toxicity, NAC potentiated its effect on weight loss, producing an additional 8% decrease in body weight (14% total).

### Open field test

**Figure 3:**
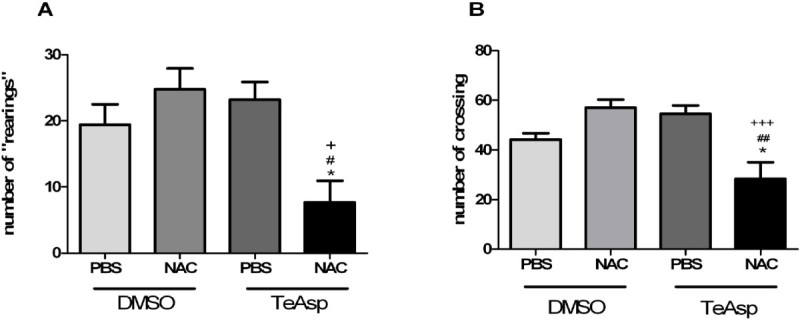
**Effects of (*S*)dimethyl 2-(3 (phenyltellanyl) propanamido) succinate (TeAsp) on open field test, number of rearings (A) and number of crossing (B) from adult mice.** Data are reported as mean ± S.E.M. of 5 animals per group. One way ANOVA followed by Newman-Keuls Multiple Comparison Test. * (p<0.05) represents significant differences from PBS/DMSO group; ^#^ (p<0.05) and ^##^ (p<0.01) represents significant differences from NAC/DMSO group and ^+^ (p<0.05) and ^+++^ (p<0.001) represents significant difference from PBS/TeAsp.

To assess whether the treatment with NAC and/or compound could cause changes in exploratory or locomotor behavior of animals, the mice were exposed to open field test and we observed that treatment with vehicle (PBS or DMSO), NAC or TeAsp did not cause any alterations in this behavior. However, when the mice were treated with TeAsp plus NAC, 60% decrease in locomotion and 35% decrease in exploratory behavior were observed (Figure 3).

### Antioxidant enzymes activity

Considering that the compound caused alterations on weigh gain and behavior of the animals exposed, we carried out the antioxidant enzymes activity to determine if the toxicity could be related to oxidative stress in brain and liver.

Brain and liver CAT and GPx (Table 1) activities were not altered by the treatments. Liver GR activity (Table 1) was elevated only in mice treated with TeAsp plus NAC.

**Table 1:** **Effect of (** ***S*** **)-dimethyl 2-(3-(phenyltellanyl) propanamido) succinate (TeAsp) on the activity of the antioxidant enzymes Catalase, Glutathione Peroxidase (GPx) and Glutathione Reductase (GR) from adult mice brain and liver**

Treatment	Catalase activity^a^		GPx activity^b^		GR activity^b^	
	Brain	Liver	Brain	Liver	Brain	Liver
**PBS/DMSO**	0.55 ± 0.04	4.44 ± 0.7	8.12 ± 0.6	93.0 ± 3.6	21.05 ± 0.1	38.6 ± 4.3
**NAC/DMSO**	0.66 ± 0.1	5.19 ± 0.8	9.09 ± 0.6	105.1 ± 9.2	25.58 ± 2.55	45.6 ± 3.9
**PBS/TeAsp**	0.57 ± 0.05	3.72 ± 0.5	8.47 ± 0.4	91.8 ± 2.5	18.94 ± 0.65	44.8 ± 5.1
**NAC/TeAsp**	0.64 ± 0.1	3.38 ± 0.32	7.06 ± 0.65	97.1 ± 7.2	19.862.0	62.5 ± 5.9*

Results are mean ± S.E.M., *n=* 5 per group. One way ANOVA followed by Newman-Keuls Multiple Comparison Test.

*Values significantly different from the PBS/DMSO group with p<0.05.

^a^Catalase activity is expressed as μmol H_2_O_2_/min/mg of protein.

^b^GPx activity and GR activity are expressed as nmol NADPH/min/mg of protein.

**Figure 4:**
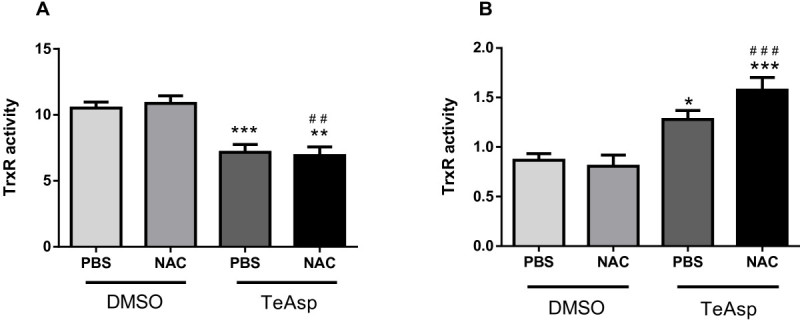
**Effects of (*S*)dimethyl 2-(3 (phenyltellanyl) propanamido) succinate (TeAsp) on TrxR activity in brain (A) and liver (B) of mice.** The results are expressed as TNB/min/mg of protein and data are reported as mean ± S.E.M. of 5 animals per group. One way ANOVA followed by Newman-Keuls Multiple Comparison Test. * (p<0.05), ** (p<0.01) or *** (p<0.001) represents significant differences from PBS/DMSO group; ^##^ (p<0.01) or ^###^ (p<0.001) represents significant differences from NAC/DMSO group.

The brain TrxR activity (Figure 4) was significantly decreased following the treatment with TeAsp (p<0.001) or TeAsp plus NAC (p<0.01). In contrast, the hepatic TrxR activity increased following the treatment with either TeAsp alone (p<0.05) or in combination with NAC (p<0.001).

### Cell viability and DNA damage

Another sign of toxicity is related to damage to DNA and cytoxicity that in this study were evaluated by comet assay and cell viability. In the mice treated in this study the administration of TeAsp alone or in combination with NAC did not cause cytotoxicity or genotoxicity to mice leukocytes (Table 2).

**Table 2:** **Cell viability and DNA damage in leukocytes of TeAsp- treated mice**

Treatment	Viability (%)	D.I.
**PBS/DMSO**	86.7±2.35	12.25±1.65
**NAC/DMSO**	86.8±3.91	11.00±1.78
**PBS/TeAsp**	91.7±2.50	13.75±4.80
**NAC/TeAsp**	94.8±0.85	9.70±3.35

Results are mean ± S.E.M., *n=* 5 per group. One way ANOVA followed by Newman-Keuls Multiple Comparison Test. Viability means the percentage of Trypan blue-negative cells.

D.I.: Damage index was calculated as follows: DI = n1 + 2n2 + 3n3 + 4n4.

Where, n1 represents the number of cells with level 1 of damage; n2, number of cells with level 2 of damage; n3, number of cells with level 3 of damage; n4, number of cells with level 4 of damage.

## Discussion

In the present study, we show that the administration of TeAsp for 7 days to mice induces significant changes on body weight, locomotor and exploratory behavior, as well as on the antioxidant enzymatic system. Previous studies have shown the antioxidant properties of Te compounds (Ávila et al. 2007; Ávila et al. 2010; Avila et al. 2012). Two previous studies investigated the effects TeAsp *in vitro* (Braga et al. 2009; Meinerz et al. 2011). However, even though presenting antioxidant properties *in vitro*, organotellurium compounds have been found to be toxic *in vivo* and *in vitro* (Sailer et al. 2004; Santos et al. 2009a, b; Funchal et al. 2011; Schiar et al. 2009; Pessoto et al. 2007). In a previous study, we found that TeAsp causes a significant mutagenic and genotoxic effect in mice when the doses administered are close to the LD_50_ (Meinerz et al. 2011). However, the mechanisms of TeAsp toxicity are still unclear. In the presented study, mice treated with TeAsp exhibited overt signs of toxicity, which included a remarkable weight loss and garlic odor exhalation. In fact, garlic odor exhalation is considered a hallmark of intoxication caused by tellurium compounds (Taylor 1996). Considering the chemistry of tellurium compounds, the oxidation of Te(II) to Te(IV) and its interaction with nucleophilic centers of biomolecules is thought to underlie the toxicity of tellurium containing organic moieties. In this context, considering that tellurium compounds can be toxic due to depletion of cellular thiol groups (Deuticke et al. 1992), we attempted to reduce TeAsp toxicity by using the thiol donor (NAC). There is abundant evidence regarding the use of NAC as an antioxidant. It is a thiol-containing molecule that has been widely used for promoting GSH synthesis (Moldeus et al. 1986; Sathish et al. 2011; Atkuri et al. 2007). The rationale for the use of NAC in the present study was that it would be able to protect against the deleterious effects of TeAsp. However, instead of protecting, we found that NAC potentiated the toxic effects of TeAsp.

Regarding the open-field behavior results, mice treated with TeAsp alone showed no difference compared to control animals. However, the mice treated with NAC plus TeAsp showed a significant decrease in locomotor activity and exploratory behavior. These results corroborate data from the literature that show that Te compounds usually exert toxic effects on the nervous system (Nogueira et al. 2002; Pinton et al. 2010). However, in the case of TeAsp, the toxic effect was observed only after concomitant treatment with NAC. We suggest that the thiol group of NAC could interact with the tellurium atom of TeAsp either by changing it to a more toxic compound or by forming a complex that could cause greater toxicity to cells of target organs. In fact, it was possible to observe an interaction between TeAsp and NAC by a spectroscopic approach using their absorption spectra between 200–800 nm. In that experiment, NAC shifted the absorption spectra of TeAsp between 320–380 nm (data not shown). That indicates that NAC reacts with TeAsp, a fact that might be occurring *in vivo* as well.

Antioxidant enzymes are considered to be a primary defense that prevents macromolecules from oxidative damage (Subramanian and James 2010). Alterations in enzymatic antioxidant systems are frequently used as markers of toxicity induced by a variety of organic compounds, heavy metals, aging and disease (Liu et al. 2004; Franco et al. 2009; Ling et al. 2011). The brain is especially susceptible to oxidative stress due to its elevated oxygen consumption (Noseworthy and Bray 1998). Accordingly, brain cells are equipped with an efficient antioxidant machinery, including the glutathione and thioredoxin systems, to dispose of the reactive species generated by high rates of aerobic metabolism (Dringen et al. 2005; Kudin et al. 2012). In this study, TeAsp did not alter the activity of most of the brain antioxidant enzymes tested (GR, GPx or CAT), even when concomitantly administered with NAC. The activity of the brain TrxR was, on the other hand, decreased following the treatment with TeAsp alone. According to previously published data, Te compounds can inhibit TrxR and cause cytotoxicity *in vitro*, probably due to the high affinity of Te compounds for the thiol and selenol groups found in TrxR (Engman et al. 2000; Engman et al. 2003). Inhibition of the Trx system is likely to further increase the brain’s susceptibility to oxidative stress. Recent studies have demonstrated the crucial role of this antioxidant system in performing peroxide detoxification in the brain (Kudin et al. 2012). In line with this evidence, the inhibition of brain TrxR by TeAsp observed in this study might be, at least in part, considered a potential mechanism of neurotoxicity induced by this organotelluride.

Comparable to what we observed in the brain, liver catalase was also unchanged after the mice were exposed to TeAsp. However, an opposite effect was observed for GR and TrxR activities in liver. Previous reports have shown that acute exposure to oxidants frequently increases the activity of antioxidant enzymes as an adaptive response which, consequently, reduces the damage (Hilbert and Mohsenin 1996). GR and TrxR are known to be involved in the regeneration of GSH and thioredoxin, respectively (Meister and Anderson 1983; Arnér and Holmgren 2000). Our data showed that these enzymes were increased in the liver of treated animals. This result could indicate an up-regulation of the cellular thiol regenerating systems. In this way, the thiol oxidizing effect of TeAsp, as well as other organotellurides (Meinerz et al. 2011), could trigger a compensatory response to TeAsp-induced hepatic GSH and Trx(SH)_2_ oxidation.

The NF-E2-related factor 2 (Nrf2) is thought to be a pivotal regulator of the ARE-driven cellular defense against oxidative stress and its regulation appears to be cell specific (Lee et al. 2005). This transcription factor binds to the “antioxidant responsive element” – ARE (Nrf2-ARE pathway) and has been shown to regulate the expression of several antioxidant proteins such as glutathione-S-transferase, GPX, GR, SOD, CAT and the thioredoxin system (Tanito et al. 2007; Hur and Gray 2011; Schülke et al. 2012). However, the positive or negative balance of Nrf2-ARE pathway activation depends on the combination of numerous factors. Several nuclear factors are shown to bind ARE, including c-Jun, c-Fos, Nrf1, Nrf2, MafG, MafK, among others (Jaiswal 2004; Dhakshinamoorthy et al. 2005). Moreover, the downregulation of the Nrf2-ARE pathway is not well understood. In this context, Nrf2 can be inactivated by Kelch-like ECH-associated protein 1 (Keap1) in the cytoplasm (Wang and Zhang 2009). In that same context, the Bach1 transcription factor has been shown to induce negative regulation of the Nrf2-ARE driven antioxidant response (Dhakshinamoorthy et al. 2005). Taking it all into account, one could suppose that the opposing responses such as the observed in our study between brain and liver GR and TrxR may be related to alternative regulation of the ARE-driven protective pathway in different tissues.

## Conclusion

Summarizing, contrary to the hypothesized, NAC did not protect against TeAsp-induced toxicity in mice. On the contrary, NAC exacerbated the general and behavioral toxicity elicited by TeAsp. Although not addressed in this study, we may speculate that the exacerbation of TeAsp toxicity by NAC may be associated with the formation of intermediate(s) with higher toxicity (for instance, diphenyl ditelluride) or to the release of Te from the organic moiety to generate a more toxic inorganic form (Te(IV)). Additional studies are necessary to determine the precise mechanisms of TeAsp toxicity and its interaction with NAC. The elucidation of such mechanisms would contribute to understanding the events mediating the therapeutic and toxic effects of Tellurium compounds.

## Authors’ original submitted files for images

Below are the links to the authors’ original submitted files for images. Authors’ original file for figure 1 Authors’ original file for figure 2 Authors’ original file for figure 3 Authors’ original file for figure 4
