# Supplementary figures and images for: Sub-acute administration of (S)-dimethyl 2-(3-(phenyltellanyl) propanamido) succinate induces toxicity and oxidative stress in mice: unexpected effects of N-acetylcysteine
Source: Springerplus. 2013 Apr 24;2(1):182. doi: 10.1186/2193-1801-2-182 (PMC3644195; doi:10.1186/2193-1801-2-182)

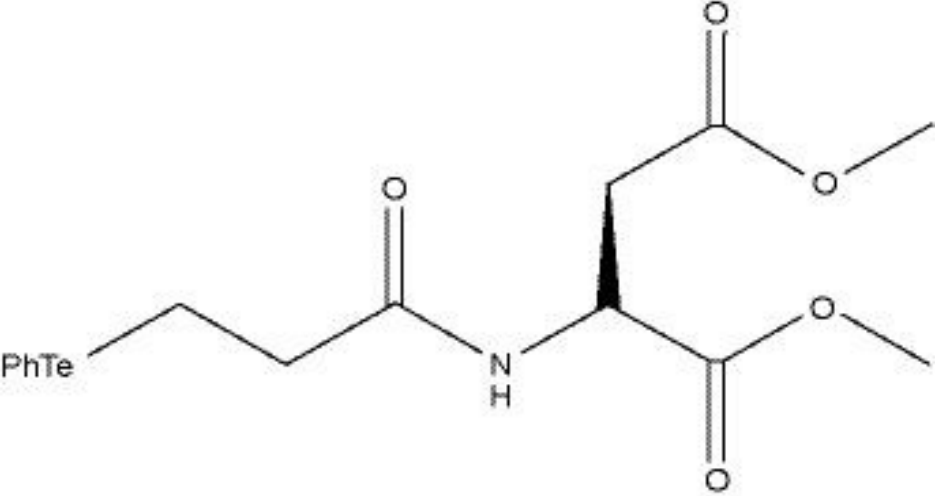

Supplement: Supplementary file 1 — Authors’ original file for figure 1 [file 40064_2012_241_MOESM1_ESM.pdf]

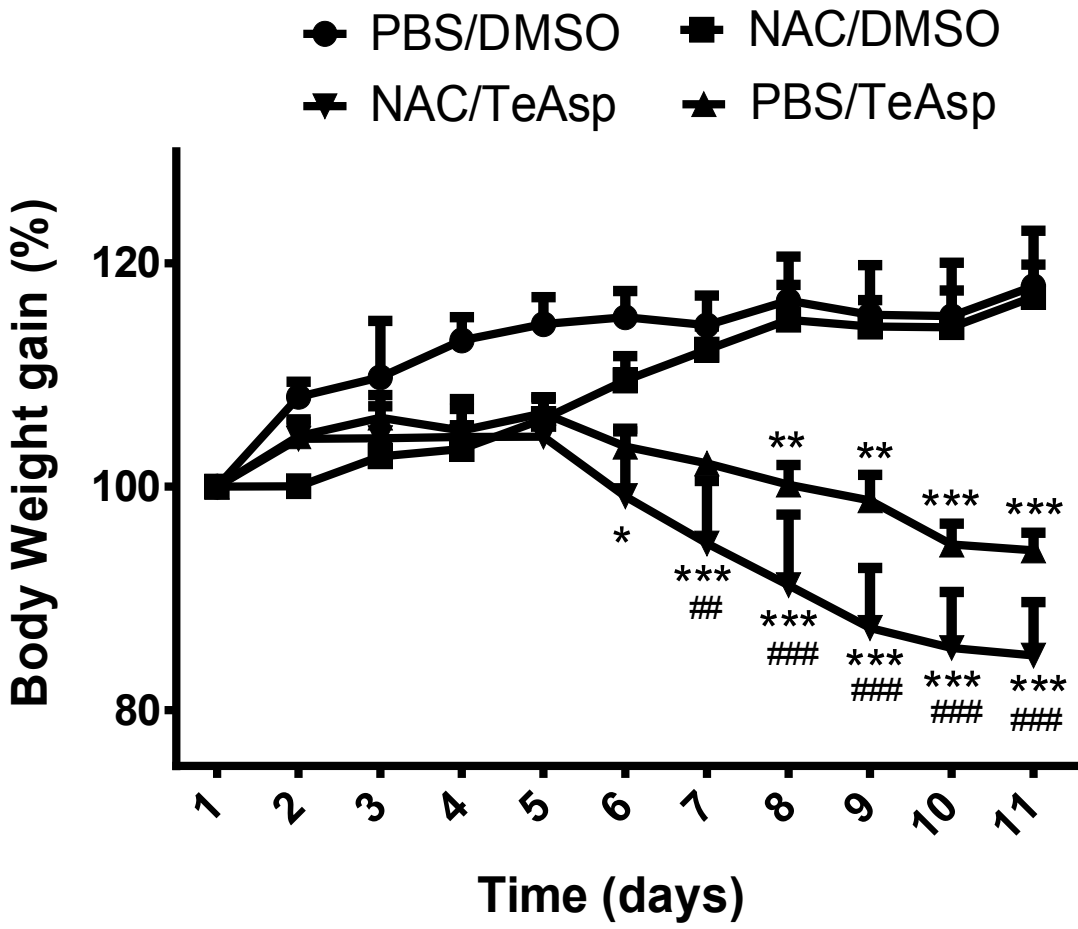

Supplement: Supplementary file 2 — Authors’ original file for figure 2 [file 40064_2012_241_MOESM2_ESM.pdf]

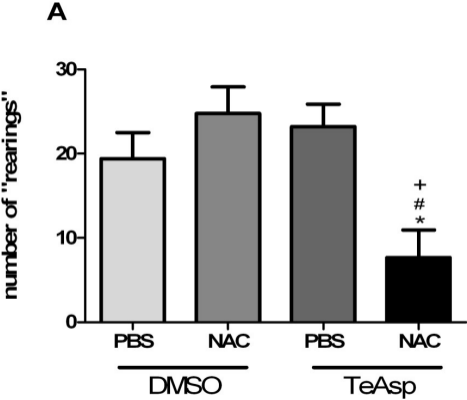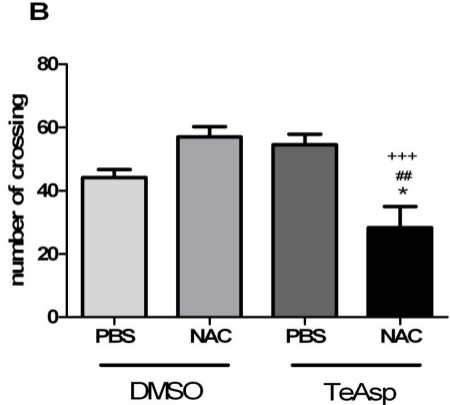

Supplement: Supplementary file 3 — Authors’ original file for figure 3 [file 40064_2012_241_MOESM3_ESM.pdf]

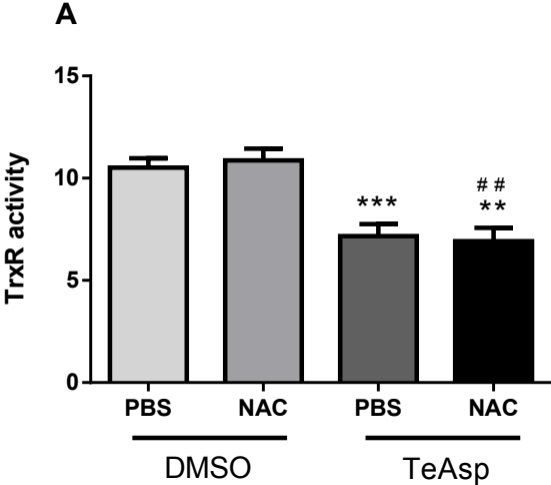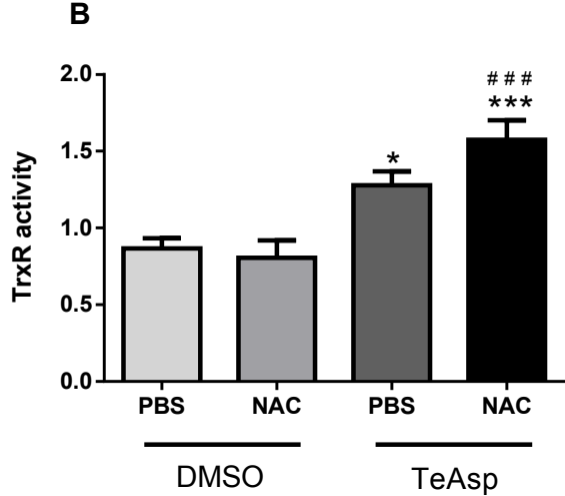

Supplement: Supplementary file 4 — Authors’ original file for figure 4 [file 40064_2012_241_MOESM4_ESM.pdf]
